# Imprinting modulates processing of visual information in the visual wulst of chicks

**DOI:** 10.1186/1471-2202-7-75

**Published:** 2006-11-14

**Authors:** Fumihiko Maekawa, Okiru Komine, Katsushige Sato, Tomoyuki Kanamatsu, Motoaki Uchimura, Kohichi Tanaka, Hiroko Ohki-Hamazaki

**Affiliations:** 1 Laboratory of Molecular Neuroscience, School of Biomedical Science and Medical Research Institute, Tokyo Medical and Dental University, Bunkyo-ku, Tokyo 113-8519, Japan; 2 Japan Society for the Promotion of Science, Chiyoda-ku, Tokyo 102-8471, Japan; 3 Department of Physiology, Division of Integrative Physiology, Jichi Medical University, Shimotsuke, Tochigi 329-0498, Japan; 4 Department of Physiology and Cell Biology, Tokyo Medical and Dental University Graduate School and Faculty of Medicine, Bunkyo-ku, Tokyo 113-8519, Japan; 5 Department of Environmental Engineering for Symbiosis, Faculty of Engineering, Soka University, Hachioji, Tokyo 192-8577, Japan; 6 "Recognition and Formation", Precursory Research for Embryonic Science and Technology (PRESTO), Japan Science and Technology Agency (JST), Kawaguchi, Saitama 332-0012, Japan

## Abstract

**Background:**

Imprinting behavior is one form of learning and memory in precocial birds. With the aim of elucidating of the neural basis for visual imprinting, we focused on visual information processing.

**Results:**

A lesion in the visual wulst, which is similar functionally to the mammalian visual cortex, caused anterograde amnesia in visual imprinting behavior. Since the color of an object was one of the important cues for imprinting, we investigated color information processing in the visual wulst. Intrinsic optical signals from the visual wulst were detected in the early posthatch period and the peak regions of responses to red, green, and blue were spatially organized from the caudal to the nasal regions in dark-reared chicks. This spatial representation of color recognition showed plastic changes, and the response pattern along the antero-posterior axis of the visual wulst altered according to the color the chick was imprinted to.

**Conclusion:**

These results indicate that the thalamofugal pathway is critical for learning the imprinting stimulus and that the visual wulst shows learning-related plasticity and may relay processed visual information to indicate the color of the imprint stimulus to the memory storage region, e.g., the intermediate medial mesopallium.

## Background

In precocial birds, such as the duck and chicken, day-old chicks rapidly form a memory of the moving object to which they were first exposed, and show a preference for it by chasing it for at least several days. This behavior, so-called imprinting [1], has attracted much attention as a model for investigating the neural basis of memory formation and recognition linked to emotional and social behavior. The level of incorporation of radioactive uracil into RNA in the forebrain roof region was found to be significantly higher in chicks that were trained by exposing them to an imprinting stimulus [2]. Using autoradiographic techniques, a training-related increase in the incorporation of radioactive uracil or 2-deoxyglucose was observed in the intermediate medial mesopallium (IMM, nomenclature according to the Avian Brain Nomenclature Forum [3]) [4,5]. Moreover, bilateral destruction of these regions before or after imprint training impaired preferences [6]. Thus, the IMM plays major roles in the acquisition and retention of imprinting preferences.

In filial imprinting, chicks learn the ensemble of characteristics presented by the mother hen. In this case, auditory as well as visual elements of the mother hen are important cues. Chicks can also be imprinted to a visual object without any auditory stimulus, which indicates that the processing of visual information in order to encode visual characteristics that should be learned before being integrated into the IMM may be involved in the establishment of visual imprinting.

Birds have two parallel visual pathways [7-9]. In the tectofugal pathway, visual information from the retina is transmitted to the following regions in the order listed: the optic tectum, the nucleus rotundus of the dorsal thalamus, and the entopallium of the telencephalon. A lesion in this pathway results in deficits in a variety of visual discriminations [10,11]. In the thalamofugal pathway, which is another visual pathway in birds, visual information from the retina terminates in the visual wulst of the telencephalon via the nucleus dorsolateralis anterior thalami. The visual wulst has been implicated in learning processes in which visual information leads to the formation of some memory representation, such as reversal learning or navigational behavior [12-17].

In the present study, we used simplified computer-generated images on a thin-film transistor liquid crystal display (TFT-LCD), and established a system to induce visual imprinting behavior in chicks directed towards moving images. We identified the role of information processing in the visual wulst in the acquisition and retention of memory by investigating the effects of visual wulst lesions. To elucidate the extent to which the visual wulst is involved in imprinting behavior, we studied using optical recording techniques the effects of imprinting on the neural response patterns of the visual wulst to stimuli. We found that three basic colors are represented in a spatially organized manner in the visual wulst, and that this pattern exhibits plastic changes after imprinting. These results indicate that the plastic alteration of the visual wulst is a prerequisite for the establishment of imprinting behavior in chicks.

## Results

### Chicks can be imprinted to a simple moving image presented on the TFT-LCD

**Figure 1:**
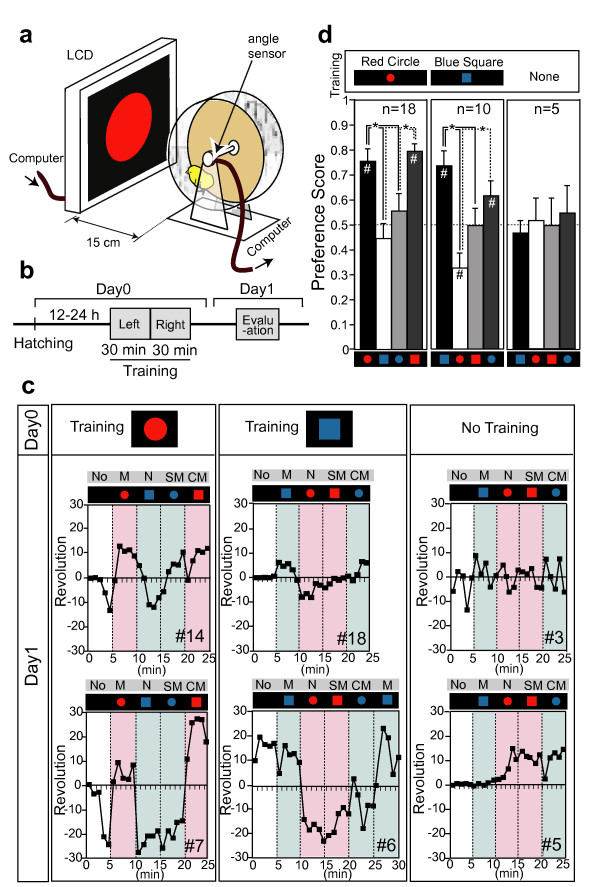
**Imprinting behavior in chicks against two-dimensional images presented on the TFT-LCD**. (**a**) Experimental apparatus used to quantify the imprinting behavior. The computer-generated image, which bounced left and right horizontally, was presented on the TFT-LCD. The chick was placed on a running wheel, and the number of forwards and backwards rotations produced by the chick was monitored. (**b**) Experimental design for training and evaluation. (**c**) Representative profiles of imprinting when chicks were trained with a red circle (left), blue square (middle) or without training (right). The numbers of revolutions per minute are plotted. A positive value shows movement towards the TFT-LCD. M, matched image; N, new image; SM, shape-matched image; CM, color-matched image. (**d**) Preference scores against various images for trained chicks (trained with a red circle or a blue square) and for in chicks without training. The preference score was calculated as: (number of revolutions towards the TFT-LCD)/(total number of revolutions during the 5-min test period). Black column, matched image; white column, new image; light-gray column, shape-matched image; gray column, color-matched image. n, number of chicks used. *, P < 0.05; #, P < 0.05.

Chicks were exposed to a computer-generated moving image (either a red circle or a blue square) on the TFT-LCD on the day of hatching (post-hatching day 0, PD0) in the training sessions. On the following day, preferences for the training image as well as for novel images were assessed (Fig. 1a and 1b). After training with exposure to the red circle, the chicks approached the TFT-LCD showing the red circle on PD1 (Fig. 1c, chicks #7 and #14). In contrast, when a blue square, an image they had never seen, was presented, the chicks moved in the opposite direction to the TFT-LCD, as if to escape from it. Upon exposure to the blue circle, an image that has the same shape but a different color to the training stimulus, the approach or escape behaviors observed depended on the individual (chicks #7 and #14). With a red square, an image that has the same color but a different shape to the training stimulus, the approach behavior was observed. Similar to the preferences of these chicks, chicks that were trained with the blue square on PD0 approached the TFT-LCD that presented the blue square but escaped from the red circle (Fig. 1c, chicks #6 and #18). With the red square, the escape behavior was observed. The blue circle induced approach or mild escape behavior. Chicks that received no training moved at random (Fig. 1c, chick #3) or moved in one direction continuously (chick #5), independent of the images presented.

Figure 1d shows the mean preference score (PS) for each 5-min presentation period. For the chicks subjected to training with the red circle or blue square on PD0, the mean PS varied significantly between the presented images [F(3,68) = 9.978, p < 0.001, and F(3,36) = 8.467, p < 0.001, respectively]. The mean PS for the training images was >0.7 for both groups (p < 0.05 compared to the chance level). In contrast, the mean PS for the new stimulus was <0.5 (p < 0.05 compared to the chance level in the blue-square-trained group). For the shape-matched image, the mean PS was only 0.5. For the color-matched image, the mean PS was about 0.8 or 0.6 (p < 0.05 compared to the chance level) when training was performed with the red circle or blue square, respectively. For the chicks trained with the red circle, the mean PS values for both the training and color-matched images were significantly higher than those for the new and shape-matched images (p < 0.01), whereas for chicks trained with the blue square, the mean PS values for both the training and color-matched images were significantly higher only for the new image (p < 0.01). In these chicks, the mean PS for the training image was significantly higher than that for the shape-matched image as well (p < 0.01). No significant difference in the mean PS was observed for chicks without training [F(3,16) = 0.145].

### Acquisition of imprinting is disrupted after generation of the visual wulst lesion

**Figure 2:**
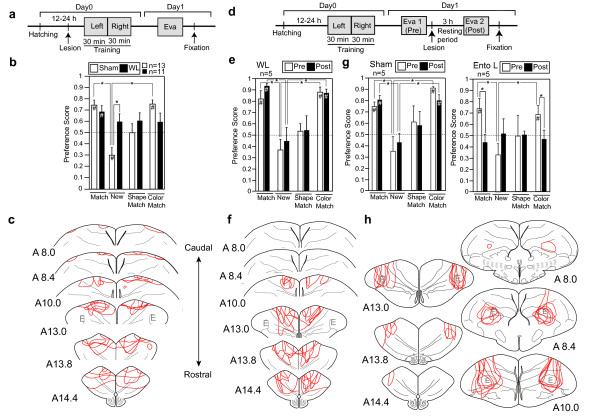
**Effects of visual wulst and entopallium lesions on imprinting behavior**. (**a**) Effects of visual wulst lesions on the acquisition of imprinting. The lesion was made before the training. Eva, evaluation. (**b**) Preference scores for various images in the sham-lesioned and visual wulst-lesioned (WL) groups. In the lesioned group, preference scores for the new image remain high. n, number of chicks used. *, P < 0.05; #, P < 0.05. (**c**) Reconstruction of damage (red lines) in the visual wulst lesioned group shown in **b**. Representative data from five chicks are presented. The numbers indicate the co-ordinates of the antero-posterior axis in the atlas [50]. E, entopallium. (**d**) Experimental design to investigate the effects of visual wulst (**e **and **f**) or entopallium (**g **and **h**) lesions after the establishment of imprinting behavior. (**e**) Preference scores during evaluations 1 (Pre) and 2 (Post) in visual wulst-lesioned chicks. Visual wulst lesioning after training and evaluation has no effect on performance. n, number of chicks used. *, P < 0.05; #, P < 0.05. (**f**) Reconstruction of lesion (red lines) in the visual wulst-lesioned (WL) group (n = 5) shown in **e**. (**g**) Preference score for each image in the sham-lesioned (left) and entopallium-lesioned (right) chicks. The sham lesion does not affect imprinting performance, while the entopallium lesion abolishes the preferences for matched and color-matched images. n, number of chicks used.*, P < 0.05; #, P < 0.05. (**h**) The extent of lesioning in the entopallium-lesioned group is shown by red lines (n = 5).

The visual wulst was destroyed before the training session, to investigate its involvement in the learning process of imprinting behavior (Fig. 2a). For chicks that received the visual wulst lesion on PD0 and that were subsequently trained with the blue square, preference for the training stimulus was not observed in this evaluation (Fig. 2b). Most of the chicks showed approach behaviors to all the presented images, regardless of shape or color. Differences in the PS for various images were not detected in the visual wulst-lesioned group, although significant differences were observed in the sham-lesioned group. A significant interaction was detected between the operation and the presented images [F(3, 88) = 4.894, p < 0.005]. The mean PS for the new image increased significantly with lesion generation (p < 0.01). The destroyed area was determined by dorsal inspection and coronal sections (Fig. 2c). It covered the apical part of the hyperpallium, the interstitial part of the hyperpallium apicale, and the intercalated as well as densocellular parts of the hyperpallium. We conclude from these results that chicks are unable to learn the imprinting stimulus in the absence of a functioning visual wulst.

### Effect of the visual wulst lesion after the establishment of imprinting behavior

The effect of bilateral ablation of the visual wulst after the establishment of imprinting behavior (Fig. 2d) is shown in Figure 2e. The mean PS after ablation was comparable to that before ablation [F(3,32) = 0.255], which implies that the wulst lesion does not affect imprinting behavior that is already established. The apical part of the hyperpallium, the interstitial part of the hyperpallium apicale, and the intercalated as well as densocellular parts of the hyperpallium were completely destroyed (Fig. 2f). In 2/5 chicks, the destruction slightly invaded the nidopallium.

Figure 2g shows the effect of sham or entopallium lesions after the first evaluation. In the sham-lesioned group, the operation did not alter the preferences, and the mean PS for each image after sham lesion introduction was comparable to that before introduction (Fig. 2g, left). In contrast, the preferences were removed after entopallium lesion introduction, and the mean PS values for the matched and color-matched images decreased significantly with the lesion (p < 0.01 for both), and reached <0.5 (Fig. 2g, right). In evaluation 2, a significant interaction was detected between the operation and the presented images [F(3, 32) = 3.801, p < 0.05]. The locations of the damaged regions in the entopallium-lesioned group are shown in Fig. 2h. Most of the entopallium was destroyed in all the chicks of this group. The ventral part of the lesion partly invaded the lateral striatum in 2/5 chicks. By passing the knife through the entopallium, a small amount of damage to the hyperpallium and nidopallium could not be avoided in all the chicks of this group. Two chicks with lesions that were mainly in the nidopallium just dorsal to the entopallium, but not in the entopallium, were not included in the entopallium-lesioned group (data not shown). The behavioral profiles of these two chicks before and after lesion introduction were similar to those of the sham-lesioned group (data not shown).

### Response of the visual wulst to electrical stimulation of the optic papilla, as revealed by optical imaging

**Figure 3:**
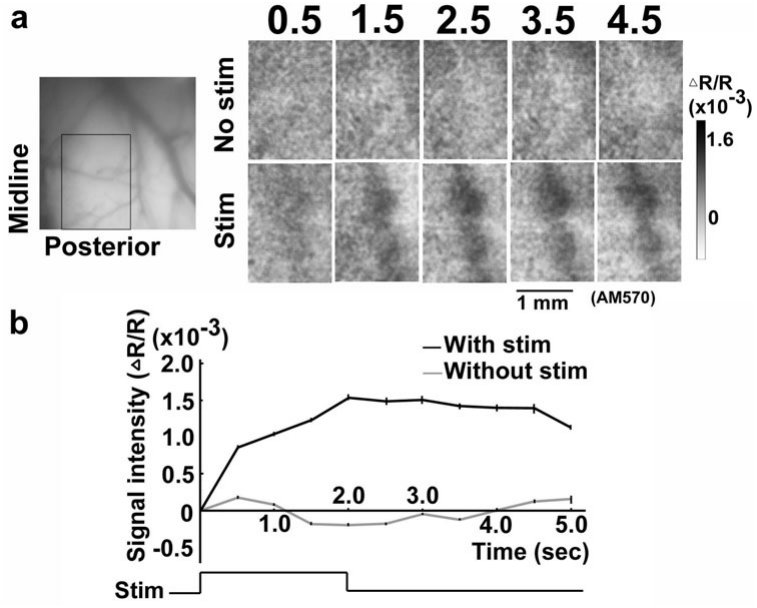
**Intrinsic optical imaging of the chick visual wulst**. (**a**) The response obtained from the right visual wulst following electrical stimulation of the left optic papilla. A vascular image of the recording area is presented in the left upper corner. The numbers in each panel indicate time (s) after stimulation. An intrinsic signal that extends from the anterior to posterior portions of the visual wulst is evoked by the electrical stimulation. (**b**) Decreasing light reflectance (signal intensity) plotted against recording time. The signal intensity peaks about 2 s after the onset of stimulation.

The responsiveness of the visual wulst was investigated using optical imaging. Initially, the optic papilla of the left eye was electrically stimulated with a bipolar electrode at 0.25 mA and 5 Hz for 2 s, and an intrinsic optical signal was obtained from the right visual wulst (n = 3). The intrinsic signal was detected as a band that expanded antero-posteriorly in the anterior medial part of the right visual wulst, surrounded by the central fissure and cerebral veins (Fig. 3a). The response peaked about 2 s after stimulus onset and lasted for several seconds (Fig. 3b). When the left optic papilla was stimulated, the response was observed predominantly in the right visual wulst, while stimulation of the right optic papilla resulted in a predominant response in the left visual wulst. This response was completely inhibited by the application of glutamate receptor inhibitors, 20 μM CNQX and 100 μM APV (data not shown).

Using a voltage-sensitive dye, we recorded the neural response of the visual wulst to optic papilla stimulation (see Additional file 1). The signal was detected in the area in which the intrinsic response was obtained. The response peaked about 70 ms after the onset of stimulation.

### Anatomical evidence for the responsive area of the visual wulst

**Figure 4:**
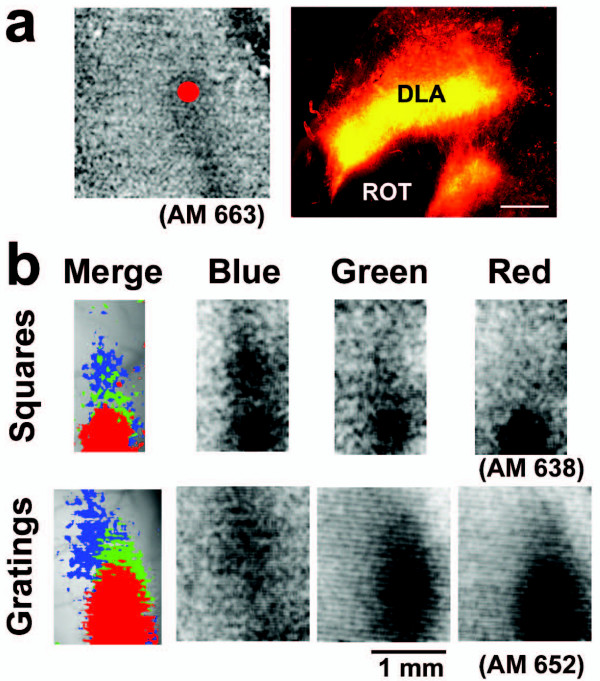
**Anatomical evidence for the area in the visual wulst that responds to the visual stimulus and intrinsic optical signals evoked in the dark-reared chicks by presentation of the colored image on the display**. (**a**) After recordings, the brain was fixed and small DiI crystals were inserted into the visual wulst region that responded to the blue square (left). The DLA (nucleus dorsolateralis anterior thalami pars lateralis) in the ipsilateral thalamus was intensely labeled retrogradely. Scale bar, 200 μm. ROT, nucleus rotundus. (**b**) Signals are evoked in the right visual wulst by the presentation of isoluminant blue, green or red squares to the left eye (upper panels). Signals evoked by the blue-black, green-black or red-black gratings that cover the full display in another chick are indicated in the lower panels. The peak regions of activation at 70% of the maximal response are colored and superimposed in the left panels. Note that the responding regions for red, green, and blue are juxtaposed sequentially from posterior to anterior.

We labeled the neurons in the visual wulst, to visualize the afferent and efferent connections of neurons that responded to the visual stimuli (n = 2). When DiI crystals were inserted into the center of the region that showed the maximum response to the blue square in the intrinsic optical recording (see the next section), neurons in the nucleus dorsolateralis anterior thalami pars lateralis in the ipsilateral thalamus were intensely labeled retrogradely (Fig. 4a). In addition, anterograde labeling was detected in the ipsilateral nucleus geniculatus lateralis pars ventralis.

### Representation of the three basic colors is spatially organized in the visual wulsts of dark-reared chicks

As we clarified in the behavioral analysis, color is an important component of the imprinting stimulus to determine image preference (Fig. 1d). Therefore, we investigated the intrinsic responses of the visual wulst neurons to colored objects in quasi-dark-reared chicks. Using the VSG system, we presented blue or red squares (as used for imprint training and evaluation) that moved horizontally across the screen. The blue and red had RGB values of 0, 0, 255 and 255, 0, 0, respectively. Each color evoked a maximum response in a different location of the visual wulst. Since these squares were not isoluminant (10.36 cd/m^2 ^for blue and 30.45 cd/m^2 ^for red), we used luminance-adjusted stimuli. Figure 4b (upper panels) shows the intrinsic optical images evoked by three isoluminant (as perceived by humans; 10.36 cd/m^2^) squares of blue, green, and red in the same visual wulst region. In the left images, the colored area corresponds to the peak regions of activation at 70% of the maximal response to each color. A comparison of the activation patterns indicates that different colors activate partially overlapping regions, and that these activations peak at different locations. In all five cases, the peak responses to red, green, and blue were organized spatially in the order of posterior to anterior, and the region that responded to red was confined to the oval-shaped restricted region, whereas the region that responded to blue always extended antero-posteriorly. To confirm that these response patterns were elicited by color differences, we presented isoluminant color gratings. In all three cases in which recordings were made successfully, a similar spatial representation of color was observed in the visual wulst (Fig. 4b, lower panels).

### Representation of colors in the visual wulst is transformed with imprinting

**Figure 5:**
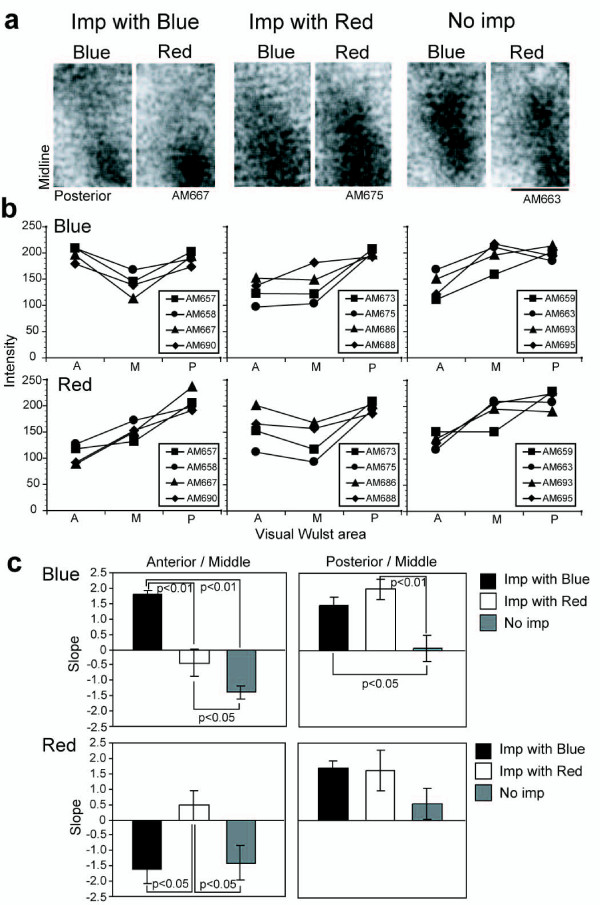
**Effect of imprinting on visually evoked intrinsic signals in the visual wulst**. (**a**) Representative intrinsic optical signals in the right visual wulst obtained from chicks imprinted with the blue square (Imp with Blue), red square (Imp with Red) or trained without image (No Imp). The blue or red square was presented on the display. The blue and red squares were the same images as those used in training and evaluation. In the blue-imprinted chicks, the intrinsic signal to blue extends from anterior to posterior, while that to red is confined to the posterior portion. In contrast, in the red-imprinted chicks, the intrinsic signal to red extends and covers the blue-responding region. Finally, in the non-imprinted chicks, the intrinsic responses to blue and red are similar, with peaks in the middle or posterior regions. AM, experimental number that designates individual chicks. Scale bar, 1 mm. (**b**) Intensities of the signals in the defined loci (see Additional files 3 and 4) evoked by blue or red squares are plotted as a function of position. In the blue-imprinted group, the response peak to blue is detected in both the anterior and posterior, while the peak response to red is only detected in the posterior position. In the red-imprinted group, the response peak to blue is detected in the posterior, while that to red is detected in the anterior and posterior, although the former is less prominent. In the no imprinting group, the response peak is observed in the middle or posterior portion, irrespective of the color of the presenting image. A, anterior; M, middle; P, posterior. (**c**) The calculated slopes between the anterior and middle and between the posterior and middle are shown. Only those chicks that were imprinted with the blue square show positive anterior/middle slopes for blue-square presentation. Similarly, only the red-imprinted chicks exhibit positive anterior/middle slopes to red. The posterior/middle slope for blue is greater for the blue- or red-imprinted chicks than for the non-imprinted chicks.

The evoked response area to the three colored squares was compared in the blue square- and red square-imprinted chicks. Chicks were trained by exposure to either blue or red squares on PD0. Imprinting was evaluated the following day. The chicks that were trained with the blue square showed an obvious preference for the blue square and circle, and were subjected to subsequent optical recordings. For these chicks, the evoked response area to blue was detected in both the anterior and posterior positions of the visual wulst, while the response area to red was restricted to the posterior portion; as a consequence, the blue-responding area entirely covered the red-responding area (Fig. 5a, left). In contrast, chicks that were trained with the red square exhibited imprinting behavior to the red square and circle. In these chicks, the blue-responding area was contracted to the posterior direction and was totally enclosed in the red-evoked area, which was enlarged anteriorly (Fig. 5a middle). We also recorded the responses of control chicks that experienced the training session without presentation of any image on the TFT-LCD (no imprinting). In these chicks, the response patterns to both blue and red squares were similar, extending from posterior to anterior with peak amplitude at the middle portion (Fig. 5a, right). The results were qualitatively similar for different criterion levels (see Additional file 2). To compare the response patterns of blue square-imprinted, red square-imprinted, and no imprinting chicks, we quantified all of the activation patterns (see Methods for details and Additional file 3 for original data). Narrow regions (5 pixels) along the antero-posterior axis were identified in each chick, and the signal intensity was plotted against this axis (see Additional file 4). Three small ROI (5 × 5 pixels) were defined at the anterior, middle, and posterior sites equidistant from each other. When the mean amplitude at each ROI was plotted as a function of position (Fig. 5b), the greatest change was observed in the response to blue in the blue-imprinted chicks. The signal amplitude of the anterior site was larger than that of the middle site, although this was not observed in the other two conditions. Similar results were obtained for the response to red in the red-imprinted chicks. The slopes were calculated and plotted, and significant differences were detected (Fig. 5c).

## Discussion

In this study, we established a system to induce the visual imprinting behavior of chicks using moving images on a TFT-LCD. In previous reports, video images were used to analyze social as well as anti-predator behaviors in chicks [18,19], and female zebra finches were shown to exhibit behavioral preferences towards males projected on a screen using a video recorder, while male finches sang songs to female images presented on a TFT-LCD [20]. Recently, Vallortigara et al. observed a preference to approach biological motion patterns presented on a display [21]. These studies indicate that birds are able to recognize images on a display as real objects or as images that resemble real animals. In accordance with this, we successfully imprinted chicks with two-dimensional images presented on a TFT-LCD. We presented two types of cues, color and shape, as key factors for discrimination. As the mean PS for the color-matched image was comparable to that for the matched image, we conclude that color is the most important factor for the discrimination of the imprinted image in our system.

We have demonstrated that the visual wulst plays an important role in processing visual stimuli, in order to transmit color and shape as imprint stimuli. It has been reported that reversal learning or sun-compass associative learning in homing pigeons is impaired after the introduction of a visual wulst lesion [12-17]. Therefore, it can be concluded that the visual wulst is involved in learning tasks that require complex judgment of visual cues, including visually guided imprinting behavior. In contrast, the entopallium lesion abolished imprinting behavior, even when it was performed after the establishment of the behavior. Our results indicate that the entopallium is indispensable for color and shape discrimination. An alternative explanation for these results is that the entopallium is important for maintaining the imprinting behavior. We favor the former explanation, as in the pigeon, the entopallium is known to be involved in various visual discriminations, such as intensity, pattern, and species [10,11].

The response properties of visual wulst neurons have been well documented by single-unit recordings of the Barn Owl. Pettigrew and Konishi [22] have reported a precise retinotopic organization, a high degree of binocular interaction, and neurons with selectivity for stimulus orientation, direction of movement, and binocular disparity, which are in common with the mammalian cortex. These properties are comparable with findings in the pigeon wulst, with the exceptions of binocular interaction and binocular disparity [23]. In the chick wulst, retinotopic organization, in which neurons that have localized receptive fields and orientation selectivity are spatially arranged, has been documented [24,25]. Recently, for the owl visual wulst, orientation preference maps were obtained using the intrinsic signal optical imaging technique [26]. In the present study, we have reported on the spatial representation of color in the chick visual wulst. Osorio et al. [27,28] have shown that domestic chicks have tetrachromatic color vision and have accurate recall of color. In the cortical area V2 of macaque monkeys, it has been reported that functional maps of colors are represented by the location of the response peak to each color [29]. Considering the fact that the sizes of the iso-orientation domains in the barn owl wulst are comparable to those found in the V2 areas of the cat and monkey [26], and that the owl visual wulst is important in contour-based form perception [30], the avian visual wulst has properties in common with the extrastriate cortical area, such as V2, in addition to V1.

Reflecting the unique layer organization of the wulst, the retinotopy of the chick is complex. The visual field is represented in the anteroposterior as well as the dorsoventral directions of the wulst [24]. Therefore, the relationship between the precise retinotopy and color-coding map in the chick visual wulst is not clear at the present time. However, considering the fact that color information along with retinotopicity can be transmitted via the tectofugal pathway, this may not represent a serious problem. Rather, the processing of visual information in the visual wulst may be specialized in a learning-related aspect.

It is documented that the projection from the thalamus to the visual wulst is lateralized functionally and anatomically, although this lateralization depends on the light condition during incubation and tasks used [31-34]. Therefore, it would be interesting to stimulate the eye opposite to the one that we used in the present study and compare the response pattern in the optical recordings.

Substantial evidence indicates that the neurons in the visual cortex are sensitive to visual experiences and exhibit plastic changes. In their pioneering work, Wiesel and Hubel [35] demonstrated that when vision from one eye was deprived during the critical period, the ocular dominance column for the non-deprived eye became dominant. This experience-dependent plasticity was also demonstrated in the orientation column [36,37]. This type of plasticity, which is dependent upon neuronal activity, has also been found in the wulst of the barn owl [38,39]. However, learning-induced plasticity in the visual cortex has only been reported for orientation selectivity [40]. In this case, specific groups of V1 cells that signal orientation differences increase their discriminatory abilities. In contrast, converging evidence of specific learning-induced plasticity in the primary auditory cortex, as well as the somatosensory cortex has been reported. The first study concerning the plasticity of the frequency receptive fields in the primary auditory cortex used a fear-conditioning paradigm, in which neurons in the auditory cortex shifted tuning towards or to the frequency of the conditioned stimulus [41]. Moreover, in owl monkeys that performed a frequency-discrimination task, an increase in the area of representation for the trained frequency was observed [42]. Thus, learning specifically altered the tonotopic map, in which the distribution of the threshold frequency is represented across the cortex. Similar expansions of cortical representation have been demonstrated in somatotopic maps of the primary somatosensory cortex [43,44].

## Conclusion

In the present study, we reported that in the chick visual wulst, a map representing at least three colors was altered following visual experience as well as imprint learning. The area representing the training-relevant color was altered, as in the case of learning-induced cortical plasticity in the primary auditory and somatosensory cortices. Assuming that the chick visual wulst is functionally similar to the mammalian cortex, this result indicates that learning-induced map plasticity is a general phenomenon in the cortical area. However, we cannot exclude the effect of emotional changes in the imprinted chicks that could alter the responsiveness of the wulst neurons to colors. Further studies are needed to clarify this point. It has been shown that the visual wulst has a neural connection with the IMM [45,46], which is considered to be a storage site for visual imprinting information [47]. Therefore, the visual wulst shows plastic changes according to the imprinting stimulus, and relays this information before it is integrated and stored in the IMM. This plastic change in the response area to the imprinting stimulus may be indispensable for the establishment of imprinting behavior.

## Methods

### Animals

Fertilized eggs of White Leghorn chickens (*Gallus domesticus*) were obtained from a local supplier (Fukuyama Shukeijo, Hiroshima, Japan), and incubated at 37.7°C. The eggs were maintained under 0 lux in the incubator except for a brief exposure to dim light (under 10 lux) one day before hatching when we transfer them to the hatching compartment. After hatching, the chicks were placed together in the same dark incubator. They were briefly exposed to dim light (under 10 lux) when we confirmed hatching and sorted them by number. We designate this condition as quasi-constant dark. These experiments were approved by the Animal Care and Use Committee and conducted according to the Guidelines for Animal Experimentation at Tokyo Medical and Dental University.

### Imprinting apparatus

To evaluate the stimulus traits to which the chicks were imprinted, we established a system that consists of computer-generated movies on a TFT-LCD (liquid crystal display). During training and evaluation, the chicks were placed on a running wheel (42 cm in diameter, 8 cm in width) with a see-through wire net on the floor (Fig. 1a). The running wheel was connected to an angle sensor that recorded the number of forwards and backwards rotations produced by the chick. The minimum detectable sensitivity of the angle sensor was 22.5° (1/16^th ^of a revolution). The data regarding the numbers of rotations in both directions were transmitted every minute to a custom-made computer system (Muromachi Kikai, Tokyo, Japan) and stored throughout the experimental period. This apparatus was the same as that used previously [48], except for the use of the TFT-LCD and computer for data collection. The running wheel was placed in a transparent vinyl chamber (70 cm × 50 cm × 50 cm) and the temperature was maintained at about 30°C using an electrically heated carpet. The 15.7-inch TFT-LCD (FPD 1570; Gateway, San Diego, CA), with a resolution of 1280 pixels × 1024 pixels driven at a refresh rate of 60 Hz, was placed 15 cm away from the edge of the wheel. The chick in the apparatus could only see through the mesh the TFT-LCD, which was placed in front of white background paper, and the featureless ceiling of the room. An AVI-format movie, made using MovieWorks ver. 4.7 (Interactive Solutions, Inc., Pleasanton, CA), was presented by Microsoft Windows Media Player. Two types of images were used for training: a red circle 17 cm in diameter (RGB values of 255, 0, and 0) and a blue square 12.5 cm per side (RGB values of 0, 0, and 255). These images occupied visual fields of 27° × 27° and 20° × 20°, respectively. When we evaluated the chick image preferences, two additional images, a red square and blue circle, were used. The colors and dimensions of these images were the same as those mentioned above. On image presentation, each image bounced left and right horizontally on the screen at the rate of 8.7 cm/s (13.8°/s).

When the optical recording was scheduled after imprint training and evaluation, the images were presented using the device described in the optical recording session.

### Training and evaluation

Figure 1b shows the training and evaluation schedules. Developing embryos and hatched chicks were maintained in a quasi-dark condition. At 12–24 h after hatching (PD0), the chicks were exposed to a training image (either a red circle or blue square) presented on the left and right TFT-LCDs (30 min for each side). The next morning (PD1, 12–24 h after training), the chick image preferences were evaluated. During the evaluation session, the TFT-LCD was always set on the left side. After a 5-min adaptation period, the red circle, blue square, blue circle, and red square were presented sequentially every 5 min to chicks that were trained with the red circle on PD0. Similarly, the blue square, red circle, red square, and blue circle were presented sequentially to chicks that were trained with the blue square. This order of image presentation represented the matched (same as the training image), new (both shape and color different from the training image), shape-matched, and color-matched images. In some cases, presentation of the matched image was repeated at the end. For the five chicks that did not undergo the training session, the images were presented in the same order as for the chicks trained with the blue square.

### Analysis of chick image preferences

The numbers of revolutions of the running wheel towards and away from the TFT-LCD were recorded every minute. Two analyses were performed to assess imprinting behavior: (1) to generate a representative graph, the number of revolutions towards the TFT-LCD minus the number of revolutions in the opposite direction was calculated every minute and plotted. This value is shown in Figure 1c; and (2) the preference score (PS), defined as the number of revolutions towards the TFT-LCD divided by the total number of revolutions during the 5-min test period, was used to compare the preferences for each image. PS values are shown in Figure 1d and Figure 2b, e, and 2g.

### Lesions and histology

To examine the role of the visual wulst in the acquisition of imprinting behavior, visual wulst lesions were made before the training session (Fig. 2a). On PD0, eleven chicks were fixed in a stereotaxic instrument under ether anesthesia and bilateral lesions were made by aspiration with a 25-gauge needle connected to an air pump. In some cases, lesions were made with fine scissors to remove the tissues. During each operation, the cerebral blood vessels served as a landmark for the region to be destroyed. For thirteen chicks (sham-lesioned group), only the skull was opened under anesthesia. After 3–5 h, the chicks were trained, and the evaluation was conducted as usual.

Figure 2d shows the schedules of training, evaluation and lesioning to investigate the effects of lesions after the first evaluation period. On PD0, fifteen chicks were exposed to the blue square as described above. Immediately after the evaluation on PD1 (evaluation 1), the chicks were subjected to the operations. Five randomly assigned chicks were subjected to bilateral visual wulst lesions. In addition, ten chicks were subjected to bilateral entopallial or sham lesions. Under ether anesthesia, these chicks were fixed on a stereotaxic instrument (model 900; David Kopf Instruments, Tujunga, CA) that was equipped with a small bird adaptor. The beak bar was lowered 8.5 mm ventrally to the ear bar. The tip of the hook-shaped Halasz knife (1.5 mm × 1.5 mm × 0.5 mm) [49] was lowered 5 mm anteriorly to the ear bar, 3.7 mm ventrally to the surface of the skull, and 3.0 mm laterally to the midline. The knife was turned 360° in the entopallial-lesioned group, whereas the knife was not turned in the sham-lesioned group. After a 3 h-resting period, image preferences were re-examined in the second evaluation session (evaluation 2)

After this evaluation, the chicks were perfused transcardially with 50 ml of 0.05 M phosphate-buffered saline, followed by 100 ml of 4% paraformaldehyde in 0.05 M phosphate buffer (pH 7.5), under deep pentobarbital anesthesia. Brains were removed and postfixed overnight in the same fixative at 4°C, and cryoprotected with 30% sucrose in 0.05 M phosphate buffer for 2–3 days at 4°C. These brains were sectioned coronally at a thickness of 50 μm using a cryostat (CM1900; Leica, Wetzlar, Germany). The sections were stained by thionin, cleared in xylene, and mounted using Entellan neu (Merck, Darmstadt, Germany). In some cases, the brains were removed under deep anesthesia and fixed with Bouin's solution. The samples were then dehydrated, cleared in xylene, and embedded in paraffin. Paraffin sections (7-μm thick) were deparaffinized, stained with thionin, and treated as above. The damage location was determined and mapped according to a stereotaxic atlas of the chick brain [50].

### Neuronal labeling using DiI

Small crystals of DiI (1,1'-dioctadecyl-3,3,3',3'-tetramethylindocarbocyanine perchlorate; Molecular Probes, Eugene, OR) were inserted into the visual wulst of the 4% paraformaldehyde-fixed brains. The brains were stored in the fixative at 37°C for several months, then rinsed in phosphate-buffered saline, and sectioned at 50-μm thickness using a vibratome. Sections were mounted on glass slides, coverslipped with an aqueous medium that contained glycerol, and examined under a fluorescence microscope (DMRA; Leica).

### Optical recordings

Under urethane anesthesia (40 mg/bird), the chicks were fixed on a stereotaxic instrument (model 900, David Kopf Instruments) that was equipped with a small bird adaptor. Body temperature was monitored continuously and maintained at 35°C with a heating mat. The skull and dura mater at the surface of the telencephalon were removed. The surface of the visual wulst was maintained in the horizontal position by adjusting the angle of the head. A plastic chamber was fixed onto the skull with dental cement (Quick Resin; Shofu, Kyoto, Japan) and filled with silicone oil (KE106; Shin-Etsu Chemical Co. Ltd., Tokyo, Japan) unless otherwise indicated. The device used for intrinsic optical imaging has been described previously [51]. Briefly, the dorsal surface of the visual wulst was illuminated by two adjustable light guides attached to 100-W tungsten-halogen lamps driven by a DC power supply (model 66184; Oriel Co., Darmstadt, Germany). The filter used for visualizing the surface of the visual wulst and its vascular pattern had a bandpass of 540 ± 30 nm, and the filters used for intrinsic imaging had a bandpass of 630 ± 30 nm (Asahi Spectra Co., Tokyo, Japan). Intrinsic imaging was performed using the differential video acquisition system Imager 2001 (Optical Imaging, Inc., Germantown, NY), via a charge-coupled device camera (CCD-5024N; Bischke Ltd., Neumünster, Germany) fitted to the tandem lens of the microscope [52]. This camera had a spatial resolution of 648 × 480 pixels. Each pixel detected the light from a square-shaped region (12 × 12 μm^2 ^and 6 × 6 μm^2^, using × 1 and × 2 magnifications, respectively) of the preparation.

For electrical stimulation of the optic nerve, the left eyeball was removed and the optic papilla was exposed. Bipolar tungsten electrodes (TN201; Unique Medical Co. Ltd., Tokyo, Japan) were attached to the left optic papilla. Pulse trains (0.25 mA, 500 μs, 5 Hz, 2 s in duration) were delivered using a pulse generator (SEN-7203; Nihon Kohden Co., Tokyo, Japan) through an isolation unit (SS-1045; Nihon Kohden). A single recording session included eight blocks. Each block consisted of four stimulation and three non-stimulation (control) trials that were interlaced randomly, with an intertrial interval of about 5 s. The data were collected over 5-s intervals and stored in a computer with the data acquisition software VDAQ (Optical Imaging).

The data were analyzed using the WinMix software (Optical Imaging). The optical data were stored as temporal sequences of eleven image frames for each trial. To exclude regions of bone or blood vessels, a rectangular subset (the region of interest; ROI) of the image data was selected, and the data inside the ROI were preserved in the new data set. The first frame of the new image data was clipped with the range of mean ± 2 mad (mean absolute deviation), and the frames that followed the first frame were clipped using the same range that was calculated for the first frame. Each of the second to eleventh frames was divided by the first frame of the same trial. In this analysis, optical reflectance changes are presented as fractional changes (deltaR/R), and the effect of uneven illumination is removed.

An *N*-methyl-D-aspartate (NMDA) receptor antagonist, DL-2-amino-5-phosphonovaleric acid (APV), and a non-NMDA receptor antagonist, 6-cyano-7-nitroquinoxaline-2,3-dione (CNQX) were purchased from Tocris Cookson (Bristol, UK). APV (100 μM) and CNQX (20 μM) were dissolved in Ringer's solution [138 mM NaCl, 5.4 mM KCl, 1.8 mM CaCl_2_, 0.5 mM MgCl_2_, 10 mM Tris-HCl, (pH 7.3)] and placed in the plastic chamber that covered the surface of the telencephalon, with the recording performed after about 90 min.

To obtain extrinsic signals, the preparation was stained for 60 min in Ringer's solution that contained the voltage-sensitive styryl dye RH414 (Molecular Probes) [53] at a concentration of 0.2 mg/ml. Excess (unbound) dye was washed away with dye-free Ringer's solution before recording. The methods we used for the multiple-site optical recording of *in vivo *electrical activity have been described in detail [54]. Briefly, light from a 300-W tungsten-halogen lamp (type JC-24V/300W; Kondo Philips Ltd., Tokyo, Japan) driven by a DC power supply (PAD35-20L; Kikusui Electronic Co., Yokohama, Japan) was collimated, rendered quasi-monochromatic with a heat cut filter and an interference filter with a transmission maximum of 520 ± 20 nm (Japan Vacuum Optics Co., Tokyo, Japan), and then reflected off a 570-nm dichroic mirror. A camera lens (a macroscopic objective) focused the light onto the preparation and collected fluorescent light. The fluorescent light was transmitted initially through the dichroic mirror and subsequently through a 610-nm long-pass emission filter. The light was then visualized using a hexagonally arranged photodiode-fiber optic camera with 464 photodiodes. The output of each detector was amplified individually, digitized, and stored in a Pentium computer (H-469II; WuTech Instruments, Gaithersburg, MD; NeuroPlex; RedShirtImaging LLC, Fairfield, CT). To visualize the vascular patterns of the cortical surface, the brain was illuminated with green light using a fiber optic system and an interference filter with a transmission maximum of 540 ± 30 nm (Asahi Spectra Co., Tokyo, Japan). The vascular image was captured with a CCD camera (C-2400; Hamamatsu Photonics Co., Hamamatsu, Japan). Electrical stimulation was applied as described above, and incident light was turned off, except during the measurement period. Fluorescent voltage-sensitive dye signals were analyzed with the NeuroPlex software (RedShirtImaging) running under IDL (Interactive Data Language; Research Systems, Boulder, CO). Eight separate recordings were summed and averaged.

To visualize the neural response to the object presented on the TFT-LCD, visual stimulus generator software (VSG, Cambridge Research Systems Ltd, Kent, UK) was used. Chicks were treated as described above, but fixed on a custom-made apparatus in which the visual field of the chick was not interrupted (Natsume, Tokyo, Japan). The 15-inch TFT-LCD (EIZO FlexScan L367; Nanao, Ishikawa, Japan) was placed 20 cm from the left eye of the chick. The colored square had sides of 8.6 cm (24° of visual field), the colored circle was 8.6 cm in diameter, and both moved horizontally with a speed of 73 mm/s (20.9°/s) for 2 s. We also used red/black, blue/black, and green/black gratings that covered the entire screen (square wave, 0.6 cycle/°, drifted at 8 cycles/s) with orientations of 45°. The colors were defined by RGB values as: red (255, 0, and 0), blue (0, 0, and 255), and green (0, 255, and 0). Therefore, the luminance values of the different colors were not identical, i.e., red (30.450 cd/m^2^), blue (10.360 cd/m^2^), and green (101.360 cd/m^2^). The CIE (Commission Internationale de l'Eclairage) -*xy *chromaticity co-ordinates of these luminance-varying colors were: red, 0.658 and 0.307; blue, 0.150 and 0.062; and green, 0.273 and 0.588. Under isoluminant (as perceived by humans) stimulus conditions, all the colors had luminance of 10.36 cd/m^2^, and the CIE-*xy *chromaticity co-ordinates of the tested colors were: red, 0.638 and 0.310; blue, 0.15 and 0.062; and green, 0.277 and 0.574. A single recording session included eight blocks. Each block consisted of three stimulation (color 1) trials, three stimulation (color 2) trials, and three non-stimulation (control) trials interlaced randomly, with an intertrial interval of about 5 s. The data were collected as described above. Each of the second to eleventh frames was divided by the first frame in the same trial. To visualize the excitation patterns for different threshold levels, the images taken 3.5 s after the onset of the stimulus were divided by the first pre-stimulus frame and processed with a 3 × 3 Gaussian filter. The same region of interest was chosen for the same animal. Using the Scion Image software (Scion Corp., Frederick, MD), each image was processed with the threshold option, and assigned thresholds of 80%, 70%, 60%, and 50% of the darkest pixel level. The peak regions of activation at more than 70% of the maximal response to each color were painted using the Adobe Photoshop software (Fig. 4). The peak of activation was defined as the single pixel with the greatest post-/pre-ratio value. To quantify each activation patterns, we first determined the antero-posterior axis. With the aid of the Adobe Photoshop software, the image was turned to adjust its antero-posterior axis to the vertical line of the frame, and then an area of 5 pixels in width that encompassed the antero-posterior axis was excised. The mean intensity of the 5 pixels (width of the defined area) was plotted against the antero-posterior position (see Additional file 4). Three loci were defined along this axis: two (the anterior and posterior) loci at the edge of the activation area and the third locus in the middle. In the case for which the most anterior activation area was not evident, the middle and posterior loci were determined initially, and the area at the same distance but in the opposite direction from the middle compared to the posterior locus was defined as the anterior locus. Three loci were constant in the one animal. In these small ROI (5 × 5 pixels), the mean intensity was calculated and plotted as a function of position (Fig. 5b). The slopes between the middle and anterior points (anterior/middle), as well as between the posterior and middle points (posterior/middle) were measured (Fig. 5c). The following formulae were used: anterior/middle = (intensity at anterior - intensity at middle)/distance in pixels; and posterior/middle = (intensity at posterior - intensity at middle)/distance in pixels.

### Statistical analysis

All the data are expressed as the mean ± SEM. The number of animals used is indicated in each figure, unless it is mentioned in the text. One-way ANOVA followed by Fisher's PLSD was used to compare the PS values between images (Fig. 1d and Fig. 2b, e, and 2g). Two-way ANOVA followed by Fisher's PLSD was used to compare the PS values between sham and lesioned, or between the pre and post groups (Fig. 2b, e, and 2g). A one sample *t*-test was used to analyze the difference from the chance level (0.5), and this is indicated by # (p < 0.05) in Figs. 1 and 2. Comparisons of the slopes among the different conditions (Imp with Blue, Imp with Red, and No Imp) were analyzed by one-way ANOVA followed by Fisher's PLSD (Fig. 5c). All the statistical analyses were performed using StatView 5.0 (SAS Institute Inc., Cary, NC). Differences were considered statistically significant for p < 0.05.

## Authors' contributions

FM, KT, and HOH conceived and designed the experiments. TK and FM contributed to the set-up of the imprinting apparatus and analysis. FM carried out the behavioral experiments. FM and MU performed the experiments related to lesions. FM, OK, and KS made the optical recordings. HOH performed the neuronal labeling. FM performed the statistical analysis. FM, KS, KT, and HOH analyzed and interpreted the data. FM and HOH drafted the manuscript. All of the authors read and approved the final manuscript.

## Supplementary Material

Additional file 1 Extrinsic optical signals in the visual wulst following electrical stimulation of the left optic papilla Click here for file

Additional file 2 The evoked activity area at four threshold levels Click here for file

Additional file 3 The evoked activity area at the 70% threshold level for each condition (imprinting with a blue square, imprinting with a red square or no imprinting) Click here for file

Additional file 4 Changes in intensities along the antero-posterior axis Click here for file
