# Supplementary material for: Imprinting modulates processing of visual information in the visual wulst of chicks
Source: BMC Neurosci. 2006 Nov 14;7:75. doi: 10.1186/1471-2202-7-75 (PMC1657023; doi:10.1186/1471-2202-7-75)
Supplement: Additional file 1 — Extrinsic optical signals in the visual wulst following electrical stimulation of the left optic papilla [file 1471-2202-7-75-S1.pdf]

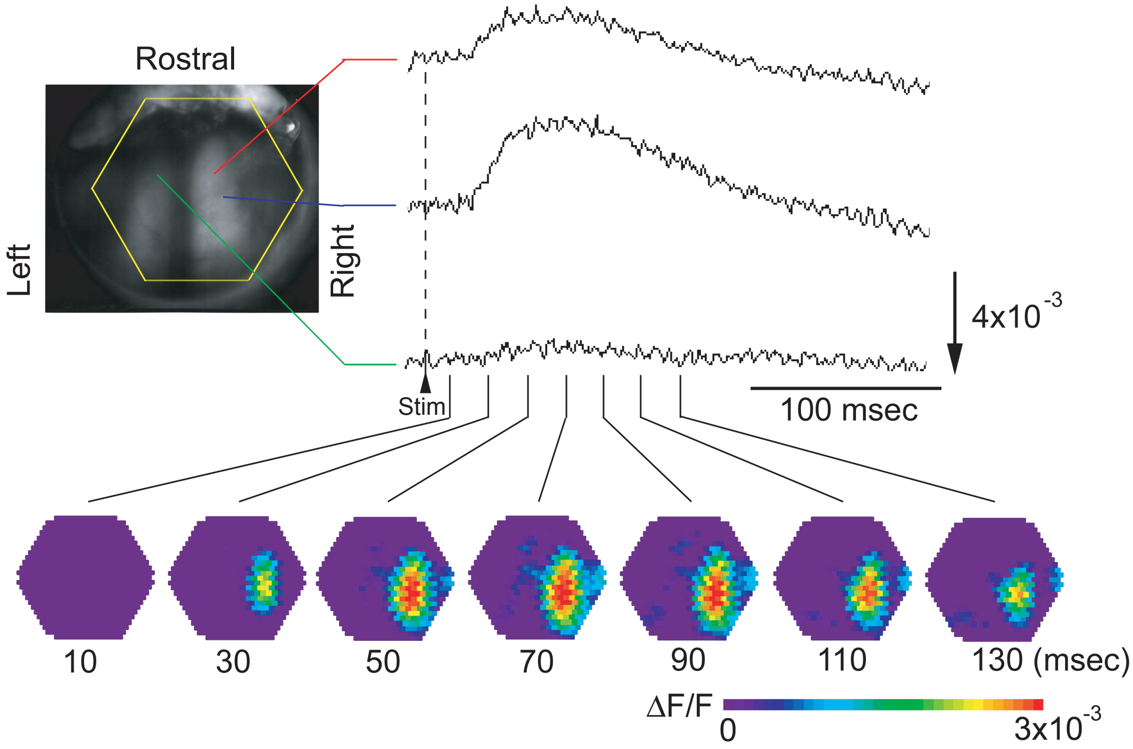

Additional File 1 Extrinsic optical signals in the visual wulst by electrical stimulation of the left optic papilla.A vascular image of the telencephalic surface, along with the hexagonal recording area, is indicated in the upper left corner. Enlarged signals from three different positions are shown on the right. Color-coded representations of the signals at 7 different timepoints are illustrated.
