# Supplementary material for: Imprinting modulates processing of visual information in the visual wulst of chicks
Source: BMC Neurosci. 2006 Nov 14;7:75. doi: 10.1186/1471-2202-7-75 (PMC1657023; doi:10.1186/1471-2202-7-75)
Supplement: Additional file 2 — The evoked activity area at four threshold levels [file 1471-2202-7-75-S2.pdf]

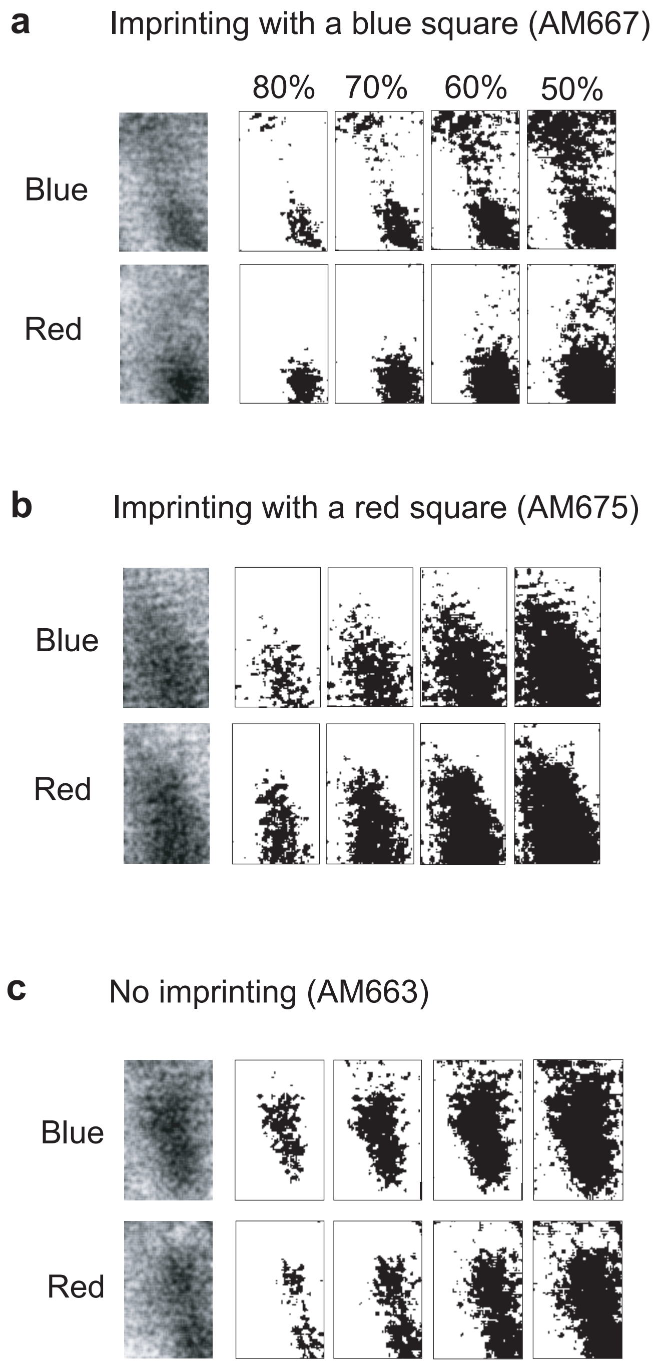

Additional File 2 The evoked activity area at four threshold levels. Response regions at indicated levels of the maximum response are shown in black. Recordings from a chick imprinted with a blue square (a), a red square (b) or no imprinting (c). Upper and lower panels in a, b and c are the responses to the blue or red square, respectively.
