# Supplementary material for: Imprinting modulates processing of visual information in the visual wulst of chicks
Source: BMC Neurosci. 2006 Nov 14;7:75. doi: 10.1186/1471-2202-7-75 (PMC1657023; doi:10.1186/1471-2202-7-75)
Supplement: Additional file 3 — The evoked activity area at the 70% threshold level for each condition (imprinting with a blue square, imprinting with a red square or no imprinting) [file 1471-2202-7-75-S3.pdf]

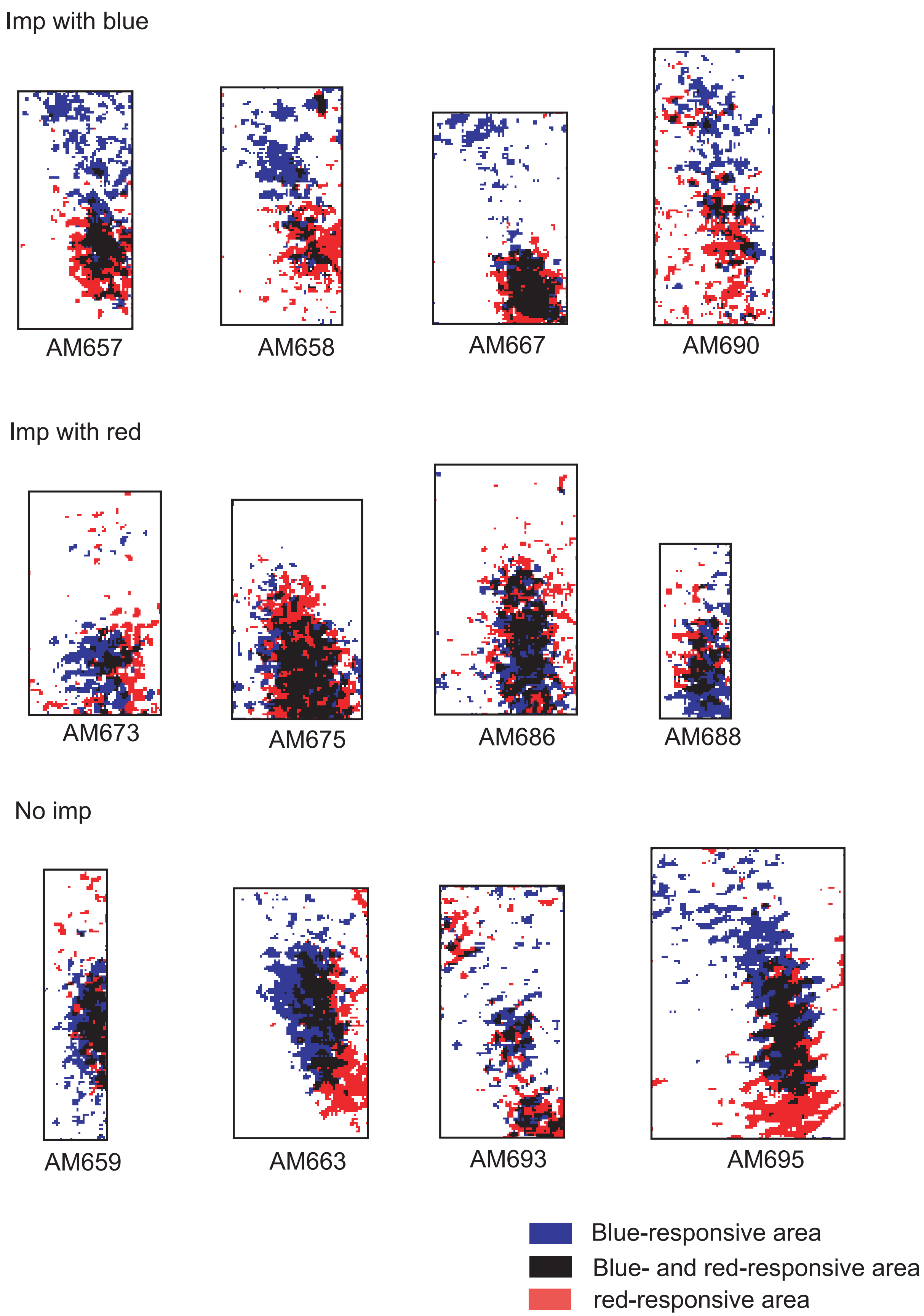

Additional File 3 The evoked activity area at 70% threshold levels in each condition (imprinting with a blue square, a red square or no imprinting).
