# Supplementary material for: Imprinting modulates processing of visual information in the visual wulst of chicks
Source: BMC Neurosci. 2006 Nov 14;7:75. doi: 10.1186/1471-2202-7-75 (PMC1657023; doi:10.1186/1471-2202-7-75)
Supplement: Additional file 4 — Changes in intensities along the antero-posterior axis [file 1471-2202-7-75-S4.pdf]

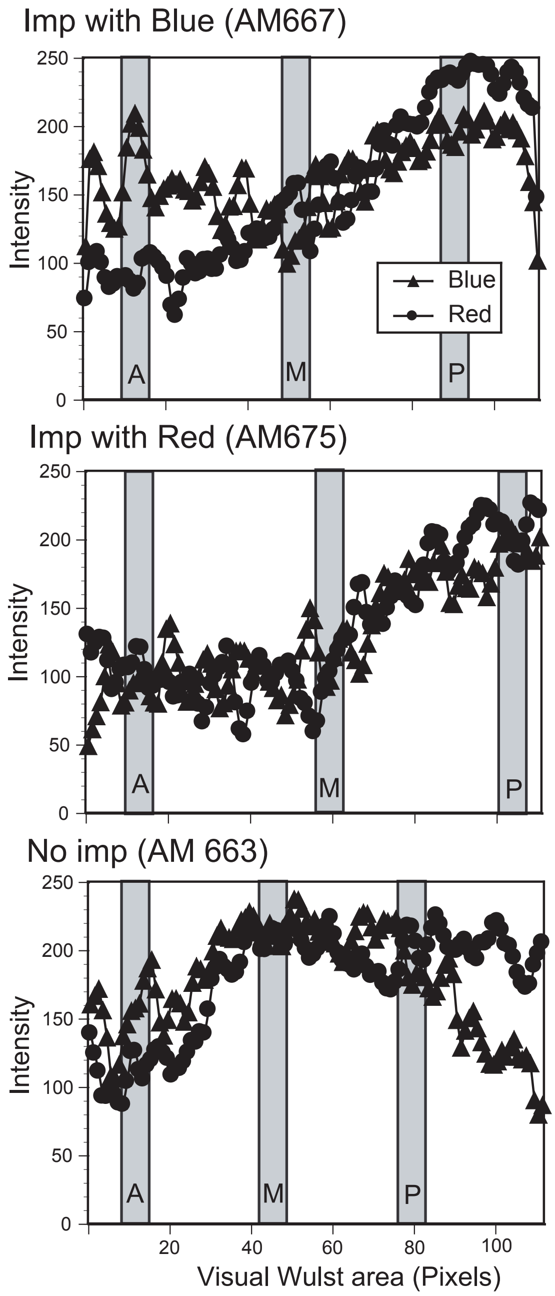

Additional File 4 Changes in intensity along antero-posterior axis. The examples of responses in each condition (imprinting with a blue square, a red square or no imprinting) were shown. Horizontal axis indicates the area along antero-posterior axis (anterior is the left). Shaded area indicates the ROI corresponding to the anterior(A), middle(M) and posterior(P) loci selected for the slope measure.
